# Analysis of Genome-Wide Association Studies with Multiple Outcomes Using Penalization

**DOI:** 10.1371/journal.pone.0051198

**Published:** 2012-12-14

**Authors:** Jin Liu, Jian Huang, Shuangge Ma

**Affiliations:** 1 Department of Biostatistics, School of Public Health, Yale University, New Haven, Connecticut, United States of America; 2 Department of Statistics & Actuarial Science, Department of Biostatistics, University of Iowa, Iowa City, Iowa, United States of America; Harvard Medical School, United States of America

## Abstract

Genome-wide association studies have been extensively conducted, searching for markers for biologically meaningful outcomes and phenotypes. Penalization methods have been adopted in the analysis of the joint effects of a large number of SNPs (single nucleotide polymorphisms) and marker identification. This study is partly motivated by the analysis of heterogeneous stock mice dataset, in which multiple correlated phenotypes and a large number of SNPs are available. Existing penalization methods designed to analyze a single response variable cannot accommodate the correlation among multiple response variables. With multiple response variables sharing the same set of markers, joint modeling is first employed to accommodate the correlation. The group Lasso approach is adopted to select markers associated with all the outcome variables. An efficient computational algorithm is developed. Simulation study and analysis of the heterogeneous stock mice dataset show that the proposed method can outperform existing penalization methods.

## Introduction

This study has been partly motivated by the analysis of the genetic architecture of complex traits in heterogeneous stock mice from Wellcome Trust Center. This data resource, which also includes pedigree information, was based on an advanced intercross mating among 8 inbred strains over 50 generations of random mating [1], [2], since the use of pseudorandom breeding for over 50 generations should result in an average distance between recombinants of 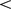
2 cM. The average linkage disequilibrium (LD), as measured by 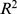 between adjacent markers, is 0.62 [3]. As with many complex mammal diseases, clinical risk factors and environmental exposures have failed to provide a comprehensive description of immunological disorders. The laboratory mouse is a key model organism for understanding gene function in mammals. Valdar et al. [1], [4] conducted a genome-wide association study and gene-environment interaction modeling to search for genetic markers possibly correlated to phenotypes. We analyze the CD4/CD8 ratio and CD4∶CD3 in this study. CD4/CD8 ratio, which is also known as the T-Lymphocyte Helper/Suppressor Profile, is a basic laboratory test in which the percentage of CD3-positive lymphocytes in the blood positive for CD4 (T helper cells) and CD8 (a class of regulatory T cells) are counted and compared. CD4∶CD3 is another clinical index for immunological diseases. Both indices are related to the diagnosis of immunological diseases. Since the indices, CD4/CD8 ratio and CD4∶CD3, are highly correlated and mechanisms behind them are related, the potentially associated genetic markers are expected to be very similar. Thus it may be more powerful to analyze the phenotypes simultaneously.

GWAS data have high dimensionality. Conventional statistical approaches analyze one SNP at a time and then adjust for multiple comparisons. Such approaches are easy to implement, however, they may contradict the fact that the development and progression of complex diseases and traits are caused by the aggregated effects of multiple SNPs. They may miss SNPs with weak marginal but strong joint effects. In the analysis of joint effects of a large number of SNPs, regularized estimation is needed. In addition, it is expected that only a subset of profiled SNPs are associated with the response variables. Thus, marker selection is needed along with estimation.

With high-dimensional data, penalization has been extensively applied for regularized estimation and variable selection. Commonly used penalization methods include Lasso, elastic net, bridge, SCAD, MCP and others. Such methods can effectively analyze data with a single response variable with interchangeable covariate effects. When there exists hierarchical structure among covariates, for example the “pathway, SNP-within-pathway” two-level structure, the “group” version of the aforementioned penalization methods have been proposed. The group penalty is usually a composite penalty. For example with group SCAD [5], the outer is the SCAD penalty, and the inner is the ridge penalty. We note that such group penalization methods are mainly used for the analysis of data with a single response variable.

In this study, our goal is to analyze data with multiple correlated response variables and conduct marker selection. In classic statistical analysis with a small number of covariates, data with multiple response variables can be accommodated under the framework of multivariate analysis of variance (MANOVA) [6] and multivariate analysis of covariance (MANCOVA). However, such methods cannot accommodate high dimensional covariates. It is possible to first apply existing penalization methods, for example Lasso, analyze each response variable separately, and then combine the analysis results using meta-analysis methods. However, such an approach ignores the correlation among response variables and hence can be less informative. Yuan and Ekici [7] introduced a nuclear norm approach encouraging the sparsity among singular values which at the same time gives shrinkage coefficient estimates and thus conducts dimension reduction and coefficient estimation simultaneously in multivariate linear models. Chen et al. [8] proposed an approach for solving reduced rank multivariate stochastic regression models.

In the heterogeneous stock mice dataset, there are multiple continuously distributed, highly correlated response variables. Under a joint modeling framework, we propose first transforming multi-response data into uni-response data following the same distribution. Then a group Lasso approach is applied to the transformed uni-response data. With two responses, the effect of one SNP needs to be represented by two regression coefficients, which naturally form a “group”. We emphasize that, unlike other group penalization studies in which one group usually corresponds to multiple covariates, here one group corresponds to a single covariate for multiple responses.

## Materials and Methods

### Analysis of multi-response data

Consider data with multiple correlated response variables. With data like the heterogeneous stock mice from Wellcome Trust Center, it is reasonable to assume that multiple responses share a certain common genetic basis, particularly the same set of susceptible SNPs. However, we note that although the response variables are correlated, they are not identical. With the inherent heterogeneity, it is not sensible to reinforce the same model with the same regression coefficients for different response variables.

Let 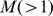 be the number of response variables, 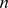 be the number of subjects, and 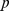 be the number of SNPs. Denote 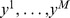 as the response variables and 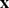 as the 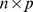 covariate matrix. For 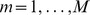, assume that 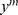 is associated with 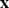 via the linear model 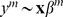, where 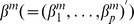 is the regression coefficient corresponding to the 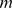
th response variable. We first transform the original data frame. For simplicity of notation, we use the same symbol 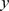 but with different subscripts for the new response variable. Although the proposed method can accommodate different covariates for distinct response variables, we assume that the same set of covariates are measured for all responses. Let 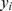 be the length-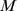 vector of response variables for the 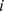
th subject, and 
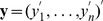
. Covariates for the 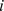
th subject have the form 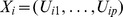 where 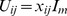. The regression coefficient vector is then 
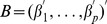
 where 
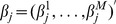
.

To better illustrate the basic features of the model settings here, consider a dataset with 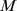 = 2 response variables and 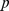 SNPs. Assume that only the first four SNPs are associated with responses. Then the coefficients may look like
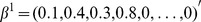


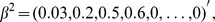
and correspondingly,
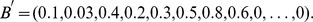
The regression coefficient 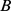 and corresponding model have the following features. First, only the first four response-associated SNPs have nonzero regression coefficients (i.e. the model is sparse). Thus marker identification amounts to identifying SNPs with non-zero regression coefficients. This strategy has been commonly used in regularized marker selection. Second, as the two response variables share the same susceptible SNPs, there is a natural grouping structure with the transformed covariates. For example, the first two regression coefficients/covariates correspond to the first SNP. Thus, they form a *group* of size two and should be selected at the same time.

Motivated by the heterogeneous stock mice dataset, we describe the proposed approach for studies with quantitative traits under linear models. The proposed approach can be extended to other types of response variables and other statistical models, as long as the joint modeling of response variables can be conducted. In a study with 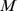 response variables, the least square loss function for transformed data can be written as
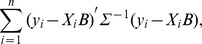
where 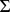 is the covariance matrix for residuals.

### Penalized estimation and marker selection

#### Penalized estimation

From definition, 
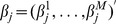
 is the coefficients for the 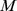 responses at the 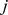
th locus. We define 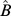 as the minimizer of the penalized least squares loss function:
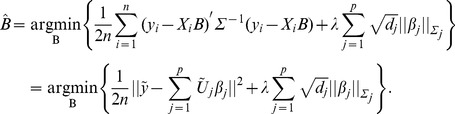
(1)Here 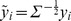, 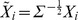, 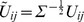, 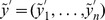, 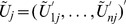, 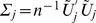, 
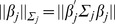
, 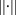 is the 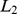 norm, and 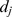 is the number of levels at the 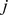
th locus (equals to 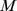 under the present setting). Note that prior to the transformation, we assume that the response follows a multivariate normal distribution. In contrast, after transformation, each element in the new response 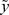 follows a univariate normal distribution. We center the response and make the grand mean equal to zero.

The proposed penalty has been motivated by the following considerations. For a given SNP locus, we treat its regression coefficients for 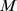 response variables as a group, so that we can evaluate its overall effects. The within-group penalty has an 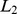 norm, and the group-level penalty has an 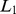 norm. Thus, the proposed penalty may have the following main properties. First, it can conduct group-level selection. Second, if a group is selected, then all members within that group are selected with non-zero estimates. But the magnitudes of regression coefficients may differ. On the other hand, if a group is not selected, all of its members are set to be zero. Such properties fit the goal of the proposed analysis.

As discussed in [9], we need to orthogonalize the transformed covariates block-wise in order to achieve computational efficiency. Write 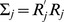 for an upper triangular matrix 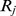 via Cholesky decomposition. Assume that 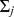 is invertible. Let 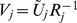 and 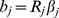, then the penalized least-squares in expression (1) becomes
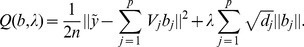
(2)If we center 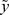, there is no need to fit for intercept for (2).

#### Computational algorithm

We use the group cyclical coordinate descent (GCD) algorithm. The GCD algorithm is a natural extension of the coordinate descent algorithm [10]. It optimizes a target function with respect to a single group parameter at a time and iteratively cycles through all group parameters until convergence. It is particularly suitable for problems as the present one which has a simple closed-form solution with a single group but lacks one with multiple groups.

The GCD algorithm proceeds as follows. For a given 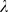,

1. Let 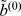 be the initial estimate. A sensible initial estimate is zero (component-wise). Initialize the vector of residuals 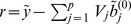 and 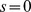.
2. For 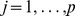, repeat the following steps:
  - Calculate the least-square estimates with respect to 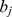


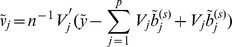


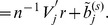

  - Compute
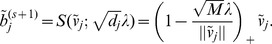
(3)
  - Update 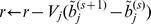.

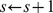
.
3. Iterate Step 2 until convergence.

Breheny and Huang [11] discussed the convergence of coordinate descent algorithms for SCAD and MCP. We now consider the GCD for group Lasso. For any given 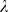, starting from an initial estimate 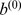, the GCD algorithm generates a sequence of updates 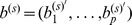, 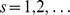, where
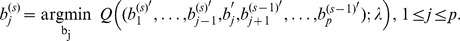
Since the sequence 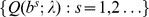 is non-increasing and bounded below by 0, it always converges. The following proposition is concerned about the convergence of 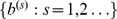.

**Proposition 1** *For any fixed* 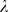, *the GCD updates* 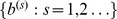 *converge to a global minimizer of the group Lasso criterion* 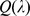 *and satisfy the inequality*

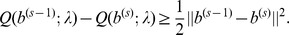



This proposition can be proved following the arguments of [12] who established the convergence of the coordinate descent algorithm for concave penalized selection methods including the Lasso.

#### Choice of tuning parameter

There are various methods that can be applied, including for example AIC, BIC, cross-validation, and generalized cross-validation. Chen and Chen [13] developed a family of extended Bayesian information criteria (EBIC) to overcome the overly liberal selection problem caused by the small-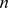-large-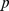 situation. Furthermore, Chen and Chen [14] established the consistency of EBIC under the generalized linear models in the small-*n*-large-*p* situation. For group Lasso, Yuan and Lin [15] proposed an approximation of the degree of freedom (DF). Here, we apply EBIC with an approximated DF to select the tuning parameter 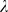. The EBIC is defined as:
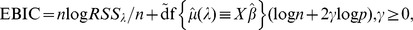
where 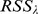 is the residual sum of squares under a fixed 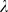. The DF for group Lasso [15] is defined as:
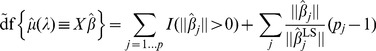
(4)where 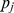 is the number of predictors in the 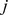
th group and 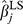 is the least-square estimate for the 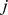
th group obtained by fitting group 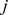 only.

Note that when 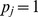 for 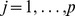, group Lasso becomes Lasso, and its DF is the number of non-zero parameters selected. Therefore, one can take Lasso as a special case of group Lasso, and so does the DF in expression (4).

#### Significance level for the selected SNPs

With penalization methods, the relevance of a covariate usually is determined by whether its regression coefficient is nonzero. As secondary analysis, it may also be of interest to compute the 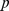 value. However, it should be noted that it is usually insensible to use both estimation magnitude (zero or nonzero) and significance level for selection.

Here, we use a multi-split method modified from the one proposed by Meinshausen et al. [17] to obtain 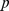-values. With linear regression, we use 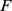-test for each group to evaluate whether there are elements in this group with significant effects. This procedure puts us in a position to obtain 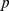-values at the group level. It is simulation-based and can adjust for multiple comparisons. The multi-split method proceeds as follows:

1. Randomly split data into two disjoint sets of equal size: 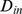 and 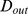.
2. Fit data in 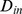 with the proposed method. Denote the set of selected groups by 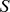.
3. Compute 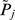, p-value for group 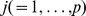, as follows:
  - If group 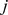 is in set 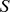, set 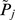 equal to the *p*-value from the *F*-test in the regular linear regression where group 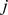 is the only group.
  - If group 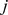 is not in set 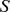, set 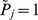.
4. Define the adjusted 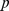-value as 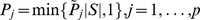, where 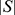 is the size of set 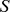.

This procedure is repeated 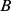 times for each group. Let 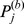 denote the adjusted *p*-value for group 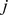 in the 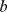
th iteration. For 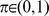, let 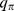 be the 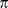-quartile of 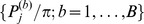. Define 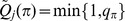. It is shown in [17] that 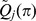 is an asymptotically correct 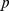-value, adjusted for multiplicity. The authors also proposed an adaptive version that selects a suitable value of quartile based on data:
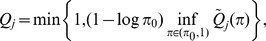
where 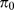 is chosen to be 0.05. It is shown that 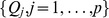, can be used for both FWER (family-wise error rate) and FDR (false discovery rate) control [17].

## Results

### Simulation studies

In simulation, we consider six different scenarios, each with 500 subjects and 5,000 or 10,000 SNPs. For each subject, we simulate two response variables. The correlation between the two responses is set to be 0.1, 0.5 or 0.9, representing weak, moderate and strong correlations. For each response variable, there are twelve SNPs with nonzero effects. Those twelve SNPs can be grouped into three clusters. Among each cluster, the correlation between two SNPs is 0.2. The correlation among SNPs not associated with response is set to be 0.2. Response-associated and noisy SNPs are independent. More specifically, the genotypes are first generated from multivariate normal distributions and then categorized into 0, 1 or 2. To mimic a SNP with equal allele frequency, we categorize genotype in a way similar to [16]. The genotype is set to be 0, 1 or 2 depending on whether 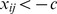, 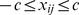 or 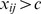, where 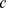 is the 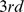-quartile of 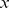. For the first response variable, the regression coefficient is
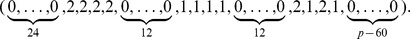
For the second response variable, the regression coefficient is
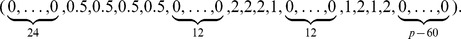
The two response variables depend on the same genotypic data and are correlated through the residuals. Clustering structure exists in this simulation.

To better gauge performance of the proposed approach, we also consider the following alternative approach. We first analyze each response variable separately using Lasso, and then combine the results by examining the overlapped SNPs. For both approaches, we apply the EBIC method described in the previous section to select the tuning parameter 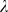. We evaluate the number of SNPs identified, number of true positives, false discovery rate (FDR) and false negative rate (FNR). In addition, estimation performance is also evaluated using SSE (sum of squared error).

Results based on 100 replicates are summarized in Table 1. Note that the true response-associated SNPs are 25–28, 41–44 and 57–60 for both responses. In total, there are 24 SNPs associated with the two responses. Table 1 shows that under all simulation scenarios, the proposed approach is able to identify almost all of the true positives, significantly more than the individual-dataset approach. The price is a few more false positives. With the proposed approach, the highest FDR is 0.18, which can be acceptable in practice. Under all scenarios, the proposed approach has significantly smaller SSEs. Taking both marker identification and estimation into consideration, we conclude that the proposed approach provides a competitive alterative to the existing individual-dataset approach. For one simulated dataset, 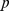-values evaluated by the multi-split method for the selected groups are presented in Table 2. It can be seen that many true positives have significant 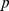-values, while all false positives have insignificant 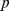-values.

**Table 1:** Simulation studies: the numbers are mean (standard deviation) based on 100 replicates.

		Combined Individual				
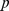	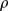	True Positive	Model Size	FDR	FNR	SSE
5000	0.1	17.60(1.99)	20.18(2.93)	0.12(0.09)	0.27(0.08)	96.89(12.67)
5000	0.5	17.80(1.82)	20.66(3.02)	0.13(0.08)	0.26(0.08)	97.85(13.51)
5000	0.9	17.66(1.72)	20.32(3.11)	0.12(0.09)	0.26(0.07)	97.39(15.61)
10000	0.1	16.78(1.64)	19.24(2.76)	0.12(0.09)	0.30(0.07)	92.76(11.16)
10000	0.5	16.84(1.80)	18.96(2.56)	0.10(0.08)	0.30(0.07)	92.44(12.57)
10000	0.9	16.70(1.79)	18.22(2.41)	0.08(0.06)	0.30(0.07)	91.37(13.68)

False discovery rate (FDR) and false negative rate (FNR) are reported together with true positives and model sizes.

**Table 2:** Multi-split 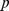-values for simulated data with all matched non-zero 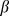
s and 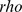 = 0.9.

	p = 5000		p = 10000	
SNP index	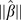	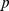 -value	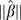	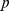 -value
25	0.293	9.3E−10	0.318	2.2E−10
26	0.270	7.3E−12	0.263	1.5E−07
27	0.263	3.1E−10	0.346	2.9E−10
28	0.251	1.1E−11	0.301	1.2E−08
41	0.264	0.054	0.182	1.000
42	0.100	1.000	0.336	0.007
43	0.245	0.006	0.249	0.019
44	0.096	1.000	0.177	0.798
57	0.107	0.004	0.093	1.000
58	0.174	1.000	0.071	1.000
59	0.183	2.3E−05	0.173	7.4E−05
60	0.089	1.000	0.094	1.000
342			0.006	1.000
2200	0.009	1.000		
3623			0.010	1.000
3920	0.013	1.000		
4177			0.004	1.000
4555			0.003	1.000
5494			0.008	1.000
5899			0.037	1.000
7156			0.020	1.000
9061			0.001	1.000
9343			0.004	1.000
9501			0.004	1.000
9884			0.013	1.000

Empty cells stand for SNPs that are not identified from the model.

With the proposed approach, it is assumed that the multiple responses of interest have exact the same set of important SNPs. Such an assumption is reasonable under some settings but too restricted under others. To get a more comprehensive understanding of the proposed approach, we also conduct simulation where the two sets of important SNPs are partially matched. In Table 3, we consider the simulation setting where 25% of the important SNPs are not matched. In Table 4, we consider the scenario with 50% unmatched important SNPs. Under both simulation scenarios, the proposed approach identifies more true positives. However, the model sizes and FDRs are much larger. Such an observation is reasonable: for a SNP associated with a single response variable, when it is identified using the proposed approach, this SNP is automatically identified for the response variable it is not associated with, creating one false positive. Thus with the proposed approach and partially matched important SNP sets, identifying more true positives inevitably leads to much larger model sizes. It is interesting to note that under all simulation scenarios, the proposed approach has significantly smaller SSEs.

**Table 3:** Simulation studies: the numbers are mean (standard deviation) based on 100 replicates.

		Combined Individual				
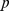	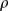	True Positive	Model Size	FDR	FNR	SSE
5000	0.1	16.96(1.92)	19.52(3.07)	0.12(0.09)	0.29(0.08)	90.82(12.20)
5000	0.5	16.96(1.82)	19.82(3.45)	0.13(0.10)	0.29(0.08)	91.35(12.19)
5000	0.9	17.10(1.67)	19.68(3.45)	0.12(0.10)	0.29(0.07)	91.63(13.66)
10000	0.1	16.06(1.73)	18.42(3.38)	0.11(0.09)	0.33(0.07)	86.30(10.36)
10000	0.5	15.92(1.70)	18.24(2.88)	0.12(0.09)	0.34(0.07)	86.13(10.90)
10000	0.9	15.96(1.75)	17.88(2.80)	0.10(0.08)	0.34(0.07)	85.43(12.50)

False discovery rate (FDR), false negative rate (FNR) and sum of squared errors (SSE) are reported together with true positives and model sizes. 25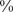 of the regression coefficients are not matched.

**Table 4:** Simulation studies: the numbers are mean (standard deviation) based on 100 replicates.

		Combined Individual				
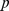	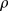	True Positive	Model Size	FDR	FNR	SSE
5000	0.1	16.94(1.89)	19.80(2.92)	0.14(0.09)	0.29(0.08)	89.29(11.00)
5000	0.5	17.00(1.92)	19.82(2.99)	0.13(0.08)	0.29(0.08)	89.67(11.41)
5000	0.9	17.08(1.90)	20.02(3.47)	0.13(0.09)	0.29(0.08)	89.94(14.40)
10000	0.1	16.26(1.55)	19.36(3.08)	0.15(0.09)	0.32(0.06)	84.41(10.06)
10000	0.5	16.20(1.58)	19.06(2.85)	0.14(0.09)	0.32(0.07)	84.24(10.48)
10000	0.9	16.16(1.60)	18.34(2.50)	0.11(0.08)	0.33(0.07)	83.38(11.16)

False discovery rate (FDR), false negative rate (FNR) and sum of squared errors (SSE) are reported together with true positives and model sizes. 50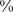 the regression coefficients are not matched.

Here we focus on the scenario with two response variables to match the data analyzed in the next section. It is possible to conduct analysis with three or more responses, which may have higher computational cost.

### Application to heterogeneous stock mice dataset

**Figure 1:**
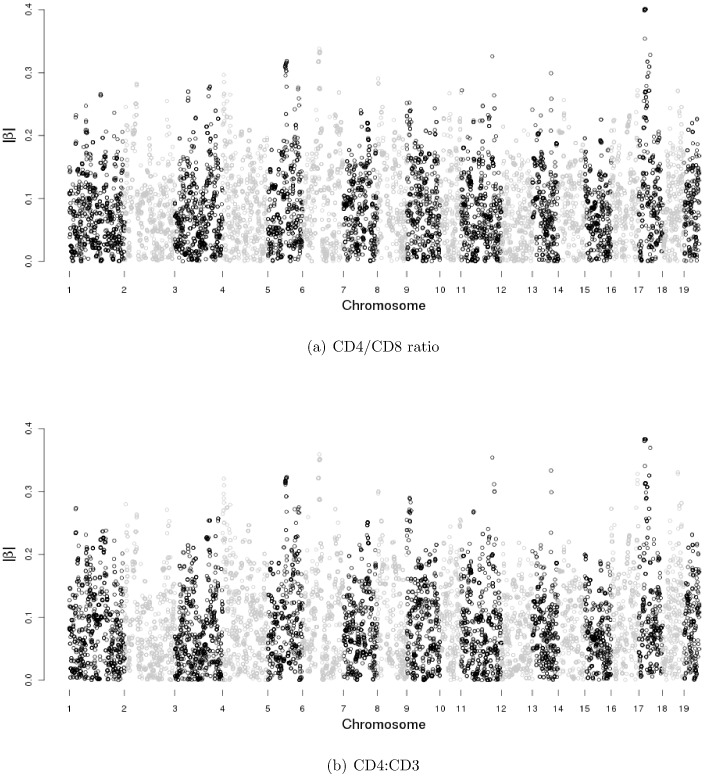
Absolute values of 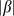
 **estimates from the simple linear regression on CD4/CD8 ratio and CD4∶CD3.**

**Figure 2:**
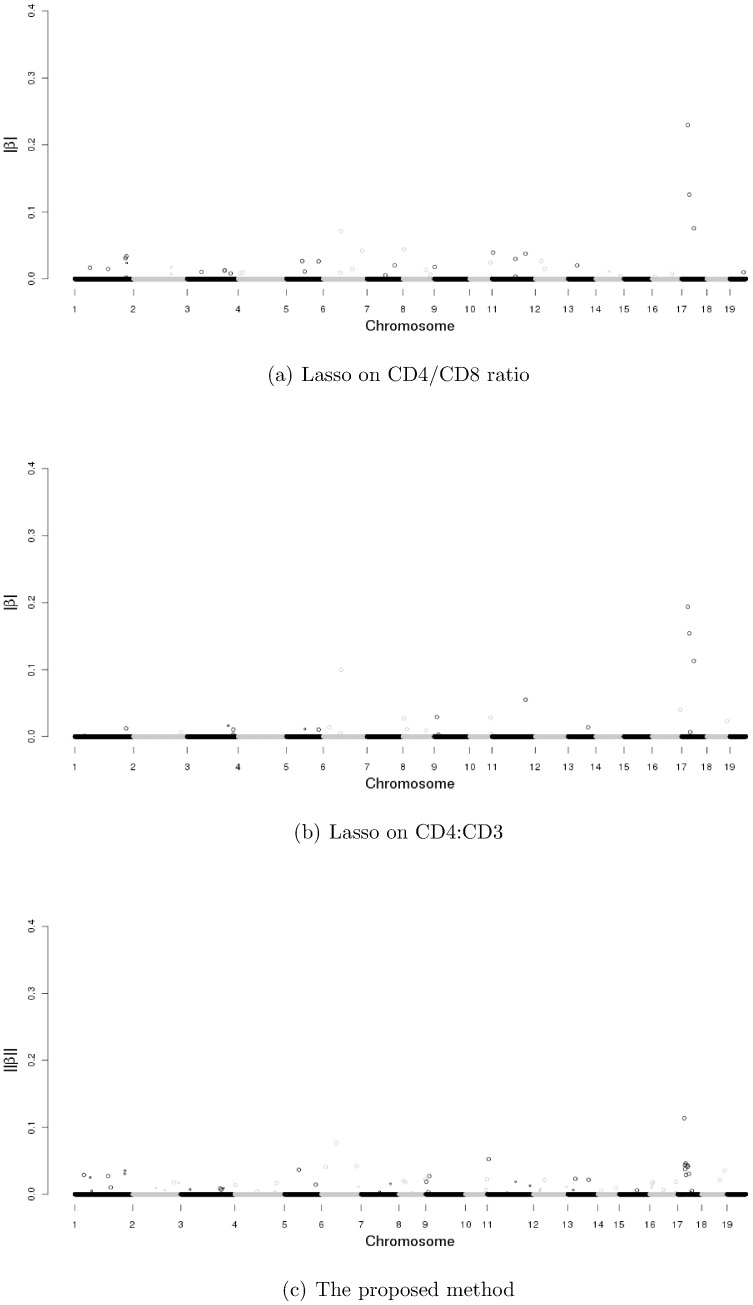
Absolute values of 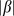
 **estimates from Lasso on CD4/CD8 ratio and CD4∶CD3 and** 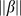 **estimates for the proposed method.** Smaller dots represent SNPs selected by the Lasso/proposed method with insignificant multi-split 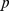-values. Larger dots represent SNPs with significant 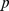-values.

The heterogeneous stock mice dataset is described in the Introduction section. We refer to the original publication for more detailed descriptions [1], [2], [4]. This dataset includes fully phenotypic records on 2,202 mice, and each was genotyped for 13,459 SNP markers. In joint modeling, SNPs with missingness cannot be included. Thus, we implement fastPHASE to impute the missingness in SNPs [18]. After deleting observations with missing phenotypes and alleles with minor allele frequency less than 0.05, there are 1,514 mice and 9,991 SNP markers in 19 autosomes. We analyze the data using three different approaches: the traditional one-SNP-at-a-time approach, analysis of individual response using Lasso, and the proposed approach. In Figure 1, we show the absolute values of 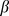 estimates from the single-SNP analysis on both CD4/CD8 ratio and CD4∶CD3. Here single-SNP analysis is conducted using a Bonferroni approach with overall 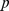-value 0.05. In Figure 2, we show the 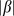 from Lasso on both phenotypes and 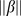 from the proposed method. In Figure 1, one can see that the signal to noise ratio is weak, and it is difficult to tell the real associated signals from background. In contrast, the signal to noise ratio is strong, and a small number of SNPs are selected by using the Lasso and proposed method. When analyzing each response separately using Lasso and multiple responses using the proposed method, we use the method described in the previous section to select the tuning parameter 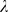. We use the multi-split method to evaluate the significance of selected SNPs. In Figure 2, the larger dots stand for the selected SNPs with significant 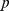-values. In Table 5, the total number of significant SNPs is summarized in the parenthesis for the Lasso on both phenotypes and the proposed method. Detailed information on the selected SNPs by the proposed method and individual Lasso methods on both CD4/CD8 ratio and CD4∶CD3 is presented in Table 6, Table 7 and Table 8, respectively. Note that there is no one-to-one correspondence between the magnitude of estimates and significance level. Such an observation is not uncommon in regression analysis. In addition, the proposed penalization approach is based on Lasso, which is known to shrink estimates towards zero. Another observation is that SNPs in high LD may have very different estimates, which is also “as expected”. In single-response analysis, Lasso has the tendency to select one out of a set of highly correlated covariates. Thus, it is possible or even likely that out of the SNPs with high LD, one may have a large estimate while others have very small or zero estimates. The numbers of selected SNPs and overlaps among the proposed method, the Lasso method and single-SNP analysis are presented in Table 5. We see that the single-SNP analysis selects a large number of SNPs. This may be due to the fact that the selection of assayed SNPs is not totally random.

**Table 5:** Number of SNPs identified, and overlap of SNPs among the proposed method, the Lasso and single-SNP analysis for heterogeneous stock mice dataset.

Method	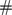 of SNPs	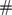 of Overlapping SNPs			
		L1*	L2**	S1***	S2****
The Proposed Method	45(38)	12	13	38	45
Lasso on M1	53(49)		10	51	53
Lasso on M2	31(28)			30	31
single-SNP analysis on M1	2964				2964
single-SNP analysis on M2	3128				

* Short for Lasso on M1.

** Short for Lasso on M2.

*** Short for single-SNP analysis on M1.

**** Short for single-SNP analysis on M2.

The number in the parenthesis is the number of SNPs with significant 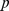-values.

**Table 6:** SNPs selected by the proposed method on both phenotypes CD4/CD8 ratio and CD4∶CD3.

SNP	Chromosome	Position	MAF	Band	Gene*	Proposed Method	
					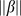	*p*-value	
rs13475794	1	32202097	0.189	1qB	Khdrbs2	0.024	1.7E−07
rs13475847	1	45969220	0.301	1qC1.1	Slc40a1	0.008	2.8E−01
rs3679459	1	120341835	0.098	1qE2.3	Clasp1	0.024	4.7E−08
rs8256197	1	130485642	0.428	1qE4	Cxcr4	0.006	3.8E−05
rs8256196	1	130485675	0.428	1qE4	Cxcr4	5.1E−15	4.3E−05
rs3682465	2	156317950	0.146	2qH1	Epb4.1l1	0.004	3.9E−07
rs3718812	3	52605874	0.155	3qC	Cog6	0.036	3.1E−08
rs3659643	3	115759847	0.205	3qG1	Extl2	2.3E−03	2.2E−06
rs6176477	3	117874757	0.259	3qG1	Snx7	4.3E−04	9.6E−06
rs13460366	4	129804978	0.137	4qD2.2	Pef1	5.5E−04	0.241
rs13477979	4	130004434	0.137	4qD2.2	Zcchc17	2.6E−15	0.332
rs13477980	4	130281564	0.137	4qD2.2	Pum1	2.4E−16	0.332
rs13478285	5	61706070	0.078	5qC3.1	G6pd2	0.015	0.003
rs3692826	5	63287018	0.078	5qC3.1	Gm17384	8.5E−16	0.004
rs6222023	5	76590704	0.397	5qC3.3	Srd5a3	0.007	2.1E−08
rs3711751	5	137393986	0.290	5qG2	4933404O12Rik	0.009	5.9E−07
rs13478656	6	21893927	0.078	6qA3.1	Ing3	0.038	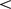 1.0E−18
rs3665567	6	71342207	0.442	6qC1	Rmnd5a	0.043	6.4E−13
rs3671932	6	134808128	0.228	6qG1	Crebl2	0.041	2.9E−07
rs3657482	7	121209199	0.458	7qF1	Rras2	0.013	0.559
rs13479673	8	30344780	0.102	8qA3	Unc5d	0.017	3.5E−08
rs33227034	8	131027085	0.480	8qE2	Nrp1	0.013	0.016
rs29634420	9	16961090	0.075	9qA2	Gm5611	0.006	0.015
rs13480141	9	36754648	0.474	9qA4	Pknox2	0.015	6.7E−07
rs13480826	10	127874456	0.194	10qD3	Rnf41	0.017	2.6E−04
rs3719526	10	127890255	0.194	10qD3	Smarcc2	1.4E−14	2.6E−04
rs3670360	11	6153674	0.107	11qA1	Ddx56	0.051	4.2E−10
rs13481186	11	100224674	0.441	11qD	Jup	0.003	2.1E−06
rs13481187	11	100513551	0.441	11qD	Zfp385c	5.5E−15	2.1E−06
rs6393715	11	111796714	0.322	11qE2	Gm11679	0.002	1.000
rs13472132	13	55515090	0.184	13qB1	Slc34a1	0.002	3.0E−05
rs3692326	13	99316615	0.143	13qD1	Gm10320	0.028	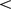 1.0E−18
rs4161101	16	10701008	0.369	16qA1	Clec16a	0.002	0.537
rs4163042	16	13142435	0.172	16qA1	Ercc4	0.001	0.005
rs3714738	16	14722893	0.091	16qA1	Si2	0.008	1.5E−03
rs4219905	16	92999911	0.348	16qC4	Runx1	0.024	3.3E−09
rs33886220	17	33354677	0.345	17qB1	Zfp955a	0.165	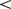 1.0E−18
rs33477985	17	33744640	0.345	17qB1	Myo1f	1.7E−15	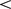 1.0E−18
rs33661797	17	35276713	0.456	17qB1	Bag6	0.038	2.4E−10
rs13482968	17	37268628	0.445	17qB1	Olfr93	0.061	1.8E−10
rs33270235	17	38311721	0.093	17qB1	Olfr134	0.011	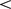 1.0E−18
rs3668036	17	45823731	0.339	17qB3	Tmem63b	0.004	7.4E−11
rs3712953	17	50402827	0.076	17qC	Dazl	0.021	6.0E−08
rs3720827	18	63449870	0.248	18qE1	Fam38b	0.020	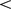 1.0E−18
rs13483449	18	77559708	0.141	18qE3	8030462N17Rik	0.023	5.1E−11

* Gene names that SNPs belong to or are closest to.

**Table 7:** SNPs selected by individual Lasso on CD4/CD8 ratio.

SNP	Chromosome	Position	MAF	Band	Gene*	Lasso	
					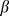	*p*-value	
rs13475847	1	45969220	0.301	1qC1.1	Slc40a1	0.017	1.4E−04
rs3727162	1	118830782	0.098	1qE2.3	Cntp5a	−0.015	8.7E−11
rs13476234	1	172771818	0.479	1qH3	Atf6	0.031	0.004
rs13476239	1	174151892	0.346	1qH3	Atp1a4	−0.002	1.9E−05
rs13476242	1	175295510	0.423	1qH3	Cadm3	−0.034	2.5E−04
rs13476251	1	176722388	0.430	1qH3	Fmn2	−0.024	1.000
rs13476764	2	127974055	0.360	2qF1	Bcl2l11	0.007	1.000
rs6411422	2	128199227	0.447	2qF1	Gm14005	0.018	1.000
rs3718812	3	52605874	0.155	3qC	Cog6	2.4E−04	8.2E−10
rs3674296	3	52738092	0.155	3qC	Cog6	−0.010	8.5E−10
rs3709732	3	117669810	0.259	3qG1	Snx7	−0.012	7.6E−12
rs6176477	3	117874757	0.259	3qG1	Snx7	0.013	7.8E−12
rs13477434	3	136689645	0.482	3qG3	Gm10955	0.008	3.7E−07
rs13477551	4	9051848	0.463	4qA1	Rps18-ps2	0.008	2.9E−09
rs13477584	4	17051798	0.353	4qA2	Gm11850	−0.009	9.7E−10
rs13478285	5	61706070	0.078	5qC3.1	G6pd2	−0.027	0.001
rs13478286	5	62201328	0.078	5qC3.1	G6pd2	3.1E−13	0.003
rs29501536	5	72711078	0.465	5qC3.2	Corin	−0.011	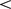 1.0E−18
rs31537882	5	72995337	0.465	5qC3.2	Cnga1	−3.4E−14	6.4E−13
rs3711751	5	137393986	0.290	5qG2	4933404O12Rik	0.026	2.29E−12
rs13478801	6	65057795	0.365	6qC1	Smarcad1	−0.009	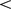 1.0E−18
rs3665567	6	71342207	0.436	6qC1	Rmnd5a	−0.072	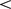 1.0E−18
rs13478941	6	103348834	0.441	6qE1	Chl1	0.015	5.6E−08
rs6334723	6	134651968	0.368	6qG1	Loh12cr1	−0.042	1.1E−12
rs13479376	7	91596873	0.074	7qD3	Gm2115	0.005	4.2E−08
rs13479465	7	120046978	0.075	7qF1	Tead1	−0.020	2.1E−04
rs13479621	8	15993378	0.451	8qA1.1	Csmd1	−0.044	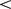 1.0E−18
rs13479930	8	97201198	0.248	8qC5	Pllp	0.013	0.001
rs6180306	8	109166165	0.074	8qD3	Cdh1	−0.006	0.002
rs29634420	9	16961090	0.075	9qA2	Gm5611	−0.018	4.2E−04
rs6280411	10	125575083	0.451	10qD3	AC153489.1	−0.024	1.11E−07
rs3701568	10	128933102	0.248	10qD3	Olfr790	0.001	1.1E−09
rs3670360	11	6153674	0.107	11qA1	Ddx56	0.039	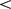 1.0E−18
rs3656583	11	64442910	0.456	11qB3	Gm12291	0.030	1.5E−09
rs6297520	11	64472210	0.456	11qB3	Gm12291	−0.003	2.5E−09
rs13481170	11	95489416	0.074	11qD	Gm11528	0.038	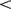 1.0E−18
rs3684699	12	28209015	0.076	12qA2	Sox11	0.027	0.001
rs13481411	12	42060667	0.071	12qB1	Immp2l	−0.015	0.001
rs13481412	12	42722660	0.071	12qB1	Immp2l	2.4E−16	0.004
rs13472132	13	55515090	0.184	13qB1	Slc34a1	0.020	3.4E−08
rs13482225	14	65324729	0.363	14qD1	Kif13b	−0.011	0.076
rs4139535	14	109988359	0.082	14qE3	Slitrk1	−0.004	0.009
rs6209981	14	110067383	0.082	14qE3	Slitrk1	−6.3E−15	0.031
rs31100152	14	110432009	0.082	14qE3	n-R5s50	7.7E−14	0.041
rs4163058	16	13269758	0.181	16qA1	Mkl2	0.003	9.3E−06
rs4163196	16	13400890	0.181	16qA1	Mkl2	1.5E−16	8.8E−05
rs4199044	16	69289859	0.449	16qC2	Speer2	0.007	1.2E−06
rs13482952	17	32937360	0.345	17qB1	Zfp811	0.230	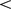 1.0E−18
rs13459151	17	33078090	0.345	17qB1	Cyp4f13	1.3E−16	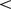 1.0E−18
rs33886220	17	33354677	0.345	17qB1	Zfp955a	−1.4E−17	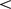 1.0E−18
rs33661797	17	35276713	0.456	17qB1	Bag6	0.126	2.1E−11
rs3712953	17	50402827	0.076	17qC	Dazl	0.076	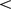 1.0E−18
rs6194426	19	50203520	0.286	19qD1	Sorcs1	0.010	1.5E−06

* Gene names that SNPs belong to or are closest to.

**Table 8:** SNPs selected by individual Lasso on CD4∶CD3.

SNP	Chromosome	Position	MAF	Band	Gene*	Lasso	
					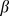	*p*-value	
rs13475794	1	32202097	0.189	1qB	Khdrbs2	−9.5E−04	1.9E−05
rs13476239	1	174151892	0.346	1qH3	Atp1a4	0.012	0.014
rs3682465	2	156317950	0.146	2qH1	Epb4.1l1	0.007	4.7E−08
rs3679962	3	127795535	0.490	3qG2	Gm10650	0.016	0.135
rs6290401	3	142297855	0.314	3qH1	Gbp2	0.003	1.2E−08
rs13477459	3	142492044	0.354	3qH1	Pkn2	0.011	2.6E−09
rs29501536	5	72711078	0.465	5qC3.2	Corin	6.1E−05	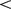 1.0E−18
rs3710735	5	73123583	0.465	5qC3.2	Txk	−1.4E−16	2.2E−12
rs6340166	5	73188279	0.465	5qC3.2	Tec	0.011	1.000
rs4225267	5	73700837	0.465	5qC3.2	Ociad1	−1.8E−16	1.000
rs3711751	5	137393986	0.290	5qG2	4933404O12Rik	−0.010	9.4E−12
rs13478656	6	21893927	0.078	6qA3.1	Ing3	0.014	2.0E−10
rs13478800	6	64766250	0.435	6qC1	Atoh1	0.005	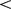 1.0E−18
rs3665567	6	71342207	0.442	6qC1	Rmnd5a	0.100	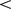 1.0E−18
rs13479621	8	15993378	0.441	8qA1.1	Csmd1	0.027	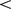 1.0E−18
rs13479673	8	30344780	0.102	8qA3	Unc5d	0.011	8.6E−09
rs13480141	9	36754648	0.474	9qA4	Pknox2	0.029	1.2E−11
rs13480153	9	40483617	0.455	9qA5.1	9030425E11Rik	−0.003	1.7E−11
rs6280411	10	125575083	0.451	10qD3	AC153489.1	0.028	8.5E−11
rs13480817	10	125932724	0.451	10qD3	AC153489.1	−1.7E−16	3.2E−10
rs29383570	10	127146595	0.420	10qD3	Myo1a	0.004	4.3E−10
rs13481170	11	95489416	0.074	11qD	Gm11528	−0.055	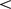 1.0E−18
rs3692326	13	99316615	0.143	13qD1	Gm10320	0.014	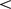 1.0E−18
rs4219905	16	92999911	0.348	16qC4	Runx1	−0.040	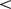 1.0E−18
rs13482952	17	32937360	0.345	17qB1	Zfp811	−0.194	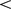 1.0E−18
rs33661797	17	35276713	0.456	17qB1	Bag6	−0.154	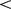 1.0E−18
rs33270235	17	38311721	0.093	17qB1	Olfr134	0.007	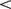 1.0E−18
rs3712953	17	50402827	0.076	17qC	Dazl	−0.113	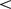 1.0E−18
rs13483448	18	77559708	0.141	18qE3	Loxhd1	−0.023	6.7E−13
rs13483449	18	77876027	0.141	18qE3	8030462N17Rik	4.8E−15	8.6E−13

* Gene names that SNPs belong to or are closest to.

With our limited knowledge on susceptibility SNPs for immunology, we are not able to objectively evaluate the biological implications of identified SNPs. As an alternative, we consider the following evaluation of prediction performance, which may provide partial information on identification performance. (a) Randomly split the sample into five parts with equal sizes; (b) Analyze four parts using the proposed approach; (c) Use the obtained model and make prediction for subjects in the left-out part; (d) Repeat Steps (b) and (c) over all five parts. For comparison, the same approach is also used to evaluate the individual Lasso approach. The prediction mean squared errors are 1.66 for the proposed approach and 2.33 for the combined Lasso. By jointly analyzing two responses, the proposed approach has better prediction performance.

## Discussion

In the study of complex diseases, it is not uncommon that a single trait cannot provide a comprehensive description, and multiple traits need to be measured. In this article, we analyze data with multiple response variables under the assumption that they have the same set of important SNPs. A penalization approach is developed for marker selection. The proposed approach can accommodate the joint effects of multiple SNPs and be more informative than single-SNP analysis. Compared with the existing approaches that analyze different traits separately, it can more effectively accommodate the correlation among traits and hence be more efficient in marker selection. Numerical studies, including simulation and analysis of the heterogeneous stock mice dataset, show satisfactory performance of the proposed approach.

The heterogeneous stock mice data have two continuous response variables with marginally normal distributions. With other types of response variables, there is a rich literature on joint modeling, which can be adopted to couple with the proposed marker selection. The proposed approach is based on the group Lasso penalty. We expect that other “group-type” penalties, such as group SCAD or group bridge, can be applied. The group Lasso is selected because of its relatively low computational cost, which is especially desirable with high-throughput data. In our numerical study, we focus on the scenario where the MAFs are not very low. When the MAFs are low, our unpublished numerical study suggests that penalization methods may not perform well because the covariate design matrix is “overly sparse”. Using penalization methods with rare variants is still being explored. Analysis of the heterogeneous stock mice data shows that the proposed approach can identify SNPs missed by single-response analysis. In addition, it has improved prediction performance. Therefore, the proposed method provides a useful alternative to the current analysis of multivariate traits in GWAS.
